# HOXA-AS2 contributes to regulatory T cell proliferation and immune tolerance in glioma through the miR-302a/KDM2A/JAG1 axis

**DOI:** 10.1038/s41419-021-04471-4

**Published:** 2022-02-18

**Authors:** Chuanhong Zhong, Bei Tao, Xianglong Li, Wei Xiang, Lilei Peng, Tangming Peng, Ligang Chen, Xiangguo Xia, Jian You, Xiaobo Yang

**Affiliations:** 1 Neurosurgery Department, the Affiliated Hospital of Southwest Medical University, 646000 Luzhou, P. R. China; 2 Sichuan Clinical Research Center for Neurosurgery, 646000 Luzhou, P. R. China; 3 Academician (Expert) Workstation of Sichuan Province, 646000 Luzhou, P. R. China; 4 Laboratory of Neurological Disease and Brain Function, 646000 Luzhou, P. R. China; 5 Rheumatism Department, the Affiliated Hospital of Southwest Medical University, 646000 Luzhou, P. R. China

**Keywords:** Neuroscience, Cell biology

## Abstract

Long non-coding RNAs (lncRNAs) have been manifested to manipulate diverse biological processes, including tumor-induced immune tolerance. Thus, we aimed in this study to identify the expression pattern of lncRNA homeobox A cluster antisense RNA 2 (HOXA-AS2) in glioma and decipher its role in immune tolerance and glioma progression. We found aberrant upregulation of lncRNA HOXA-AS2, lysine demethylase 2A (KDM2A), and jagged 1 (JAG1) and a downregulation of microRNA-302a (miR-302a) in glioma specimens. Next, RNA immunoprecipitation, chromatin immunoprecipitation, and dual-luciferase reporter gene assay demonstrated that lncRNA HOXA-AS2 upregulated KDM2A expression by preventing miR-302a from binding to its 3′untranslated region. The functional experiments suggested that lncRNA HOXA-AS2 could promote regulatory T (T_reg_) cell proliferation and immune tolerance, which might be achieved through inhibition of miR-302a and activation of KDM2A/JAG1 axis. These findings were validated in a tumor xenograft mouse model. To conclude, lncRNA HOXA-AS2 facilitates KDM2A/JAG1 expression to promote T_reg_ cell proliferation and immune tolerance in glioma by binding to miR-302a. These findings may aid in the development of novel antitumor targets.

## Introduction

Glioma is one of the most dangerous cancers, and has lamentably few treatment options [1]. Traditional approaches for glioma therapy can delay tumor progression, but have only minimal effects on patients’ dismal overall survival rates [2]. Currently, investigators are making increasing efforts to explore how one might induce antitumor immunity to kill selectively the infiltrating glioma cells without destroying healthy brain tissues [3]. Thus, a better understanding of the complex relationship between gliomas and the immune system could benefit the development of novel glioma therapies. Accumulating evidence has revealed the occurrence of certain adaptions in gliomas, such as induction of regulatory T cell (T_reg_) phenotypes and myeloid-derived suppressor cells that initiate immune tolerance, which is regarded as a mechanism of resistance to targeted therapies in diseases [4, 5] that promotes disease progression [6, 7].

Non-coding RNAs are a large and diverse group of transcripts that span the eukaryotic genome, including microRNAs (miRNAs or miRs), long non-coding RNAs (lncRNAs), and circular RNAs [8]. The lncRNAs are without protein coding capacity, despite their length exceeding 200 nucleotides [9, 10], but have been proved to play roles in diverse biological processes including the regulation of tumor immune response [11]. Zhang et al. have reported that the lncRNA known as nuclear enriched abundant transcript 1 (NEAT1) can suppress NOD-like receptor pyrin domain containing 3 (NLRP3) inflammasome to induce the tolerogenic phenotype in dendritic cells [12]. Meanwhile, other research has revealed that the lncRNA Flatr promotes immune tolerance phenotype by orchestrating forkhead box protein P3 (Foxp3) in T_reg_ cells [13]. Furthermore, the lncRNA homeobox A cluster antisense RNA (HOXA-AS) is reported as a promising therapy target and biomarker for tumors like non-small cell lung cancer (NSCLC), pancreatic cancer, and hepatocellular carcinoma [14–16]. LncRNA HOXA-AS2 expression is reportedly augmented in glioma tissues and cells, to an extent that correlates with tumor size and advanced pathological stage [17]. Previous studies unraveled that lncRNA HOXA-AS2 can upregulate IGF-2 expression to promote NSCLC cell migration and invasion [18]. Although the role of lncRNA HOXA-AS2 in progression and metastasis has been addressed in these aforementioned cancers, its potential role in glioma progression and immune tolerance remains unknown [19]. Recent research has revealed that lncRNA HOXA-AS2 regulates gene expression by acting as a scaffold for epigenetic modifiers or by sponging miRNAs [14, 19]. The miRNAs are small non-coding RNAs with a length of ~22 nucleotides, which have the capability to modulate gene expression at the post-transcriptional level and also participate in diverse biological processes [20, 21]. For example, miR-302a is a tumor suppressor that restrains glioma cell proliferative, migrating, and invasive properties by targeting Grb2-associated binding protein 2 (GAB2) [22]. In this study, we performed gain- and loss-of-function analyses to reveal in some detail the participation of the lncRNA HOXA-AS2-miR-302a axis in immune tolerance and glioma progression.

## Results

### LncRNA HOXA-AS2 was upregulated in glioma and correlated with poor outcomes of patients with glioma

**Fig. 1:**
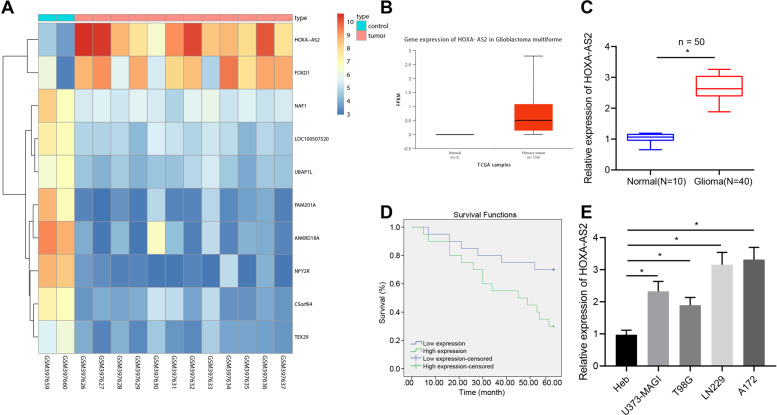
LncRNA HOXA-AS2 is highly expressed in glioma and indicates poor prognosis of patients with glioma. **A** A heat map of the expression of the top 10 differential genes in the GSE15824 dataset. **B** The expression of lncRNA HOXA-AS2 in glioma and normal samples in TCGA database. **C** LncRNA HOXA-AS2 expression in tumor tissues from glioma patients (*N* = 40) and normal brain tissues (*N* = 10) determined by RT-qPCR. **D** Correlation analysis between the lncRNA HOXA-AS2 expression and overall survival rates of patients with glioma using the Kaplan–Meier method. **E** LncRNA HOXA-AS2 expression in glioma cell lines U373-MAGI, LN229, A172, and T98G, and Heb cells determined by RT-qPCR. *p < 0.05. Data were shown as the mean ± standard deviation. Statistical comparisons were performed using unpaired *t*-test when only two groups were compared or by one-way ANOVA with Tukey’s post hoc test when more than two groups were compared. Cell experiments were repeated three times.

Analysis of the GSE15824 dataset suggested that lncRNA HOXA-AS2 was highly expressed in glioma samples, which was further verified by the UALCAN website (Fig. 1A, B). The results of RT-qPCR showed higher lncRNA HOXA-AS2 expression in tumor tissues of patients with glioma compared to control brain tissues (Fig. 1C). Meanwhile, Kaplan–Meier analysis revealed that lncRNA HOXA-AS2 expression was negatively correlated with overall survival rates of glioma patients (Fig. 1D). Consistent with the clinical data, lncRNA HOXA-AS2 expression was high in glioma cell lines U373-MAGI, LN229, A172, and T98 compared to the normal glial Heb cell line, of which, LN229 and A172 cell lines exhibited the highest expression of lncRNA HOXA-AS2 (Fig. 1E) and were therefore selected for subsequent experimentations. The above results indicated that lncRNA HOXA-AS2 was robustly induced in glioma and related to the poor prognosis of patients with glioma.

### LncRNA HOXA-AS2 silencing repressed T_reg_ cell proliferation and immune tolerance, thus arresting glioma progression

**Fig. 2:**
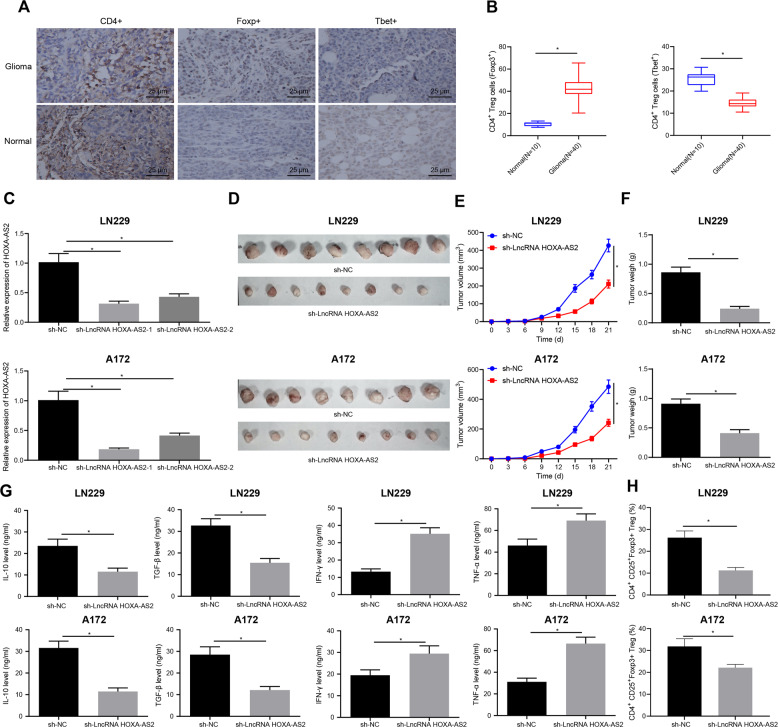
LncRNA HOXA-AS2 silencing suppresses T_reg_ cell proliferation and immune tolerance, therefore curtailing the glioma progression. **A** IHC analysis of CD4, Foxp3, and T-bet proteins in glioma tissues (*N* = 40) and normal brain tissues (*N* = 10). **B** Quantitative analysis of (**A**). **C** LncRNA HOXA-AS2 silencing efficiency in LN229 and A172 cells determined by RT-qPCR. **D** Representative images of resected tumors from BALB/c mice injected with the LN229 and A172 cells stably infected with lentivirus harboring sh-NC or sh-lncRNA HOXA-AS2 (*n* = 8). **E** Measurement of tumor volume of BALB/c mice following treatment with sh-lncRNA HOXA-AS2 (*n* = 8). **F** Measurement of tumor weight of BALB/c mice following treatment with sh-lncRNA HOXA-AS2 (*n* = 8). **G** IL-10, TGF-β, IFN-γ, and TNF-α levels in spleen tissues of BALB/c mice following treatment with sh-lncRNA HOXA-AS2 detected by ELISA. **H** Ratio of CD4^+^CD25^+^Foxp3^+^ cells in tumor tissues of BALB/c mice following treatment with sh-lncRNA HOXA-AS analyzed by flow cytometry. *p < 0.05. Data were shown as the mean ± standard deviation. Statistical comparisons were performed using unpaired *t*-test when only two groups were compared or by one-way ANOVA with Tukey’s post hoc test when more than two groups were compared. Data at different time points were compared by repeated measures ANOVA with Bonferroni post hoc test. Cell experiments were repeated three times.

The results of IHC showed that compared with the normal brain tissues, Foxp3^+^ cells (Foxp3^+^T_reg_/CD4^+^ T cells) were increased significantly in the glioma tissues while T-bet^+^ cells (T-bet^+^Th1/CD4^+^ T cells) were significantly reduced (Fig. 2A, B). RT-qPCR results displayed that lncRNA HOXA-AS2 expression was decreased by sh-lncRNA HOXA-AS2-1 and sh-lncRNA HOXA-AS2-2, and that lncRNA HOXA-AS2 expression was 69% lower after sh-lncRNA HOXA-AS2-1 transduction. sh-lncRNA HOXA-AS2-1 sequence showed better silencing efficiency (Fig. 2C) and was used for subsequent experimentations.

Furthermore, tumor volume and weight were found to be obviously reduced in BALB/c mice silencing lncRNA HOXA-AS2 (Fig. 2D–F). ELISA data presented that IL-10 and TGF-β levels were significantly diminished, while IFN-γ and TNF-α levels were augmented in the spleen tissue of BALB/c mice following silencing of lncRNA HOXA-AS2 (Fig. 2G). Flow cytometric data exhibited that lncRNA HOXA-AS2 silencing led to a significantly lower ratio of CD4^+^CD25^+^Foxp3^+^ cells in the tumor tissues. In addition, the ratio of CD4^+^CD25^+^Foxp3^+^ cells was reduced to 56.5% in the tumor tissues of BALB/c mice inoculated with LN229 cells transfected with sh-lncRNA HOXA-AS2, while it was reduced to only 29.2% in tumor tissues of BALB/c mice inoculated with A172 cells transfected with sh-lncRNA HOXA-AS2 (Fig. 2H). Collectively, these data revealed that lncRNA HOXA-AS2 knockdown could suppress T_reg_ cell proliferation and immune tolerance, thus delaying the glioma progression.

### LncRNA HOXA-AS2 facilitated KDM2A expression by binding to miR-302a

**Fig. 3:**
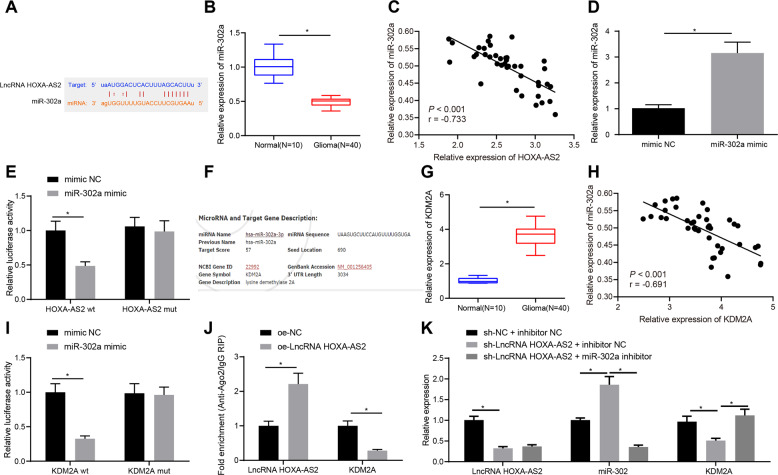
LncRNA HOXA-AS2 increases KDM2A expression by binding to miR-302a. **A** Binding sites of miR-302a on the 3′UTR of lncRNA HOXA-AS2 predicted by the DIANA TOOLS, RAID v2.0 and TargetScan databases. **B** miR-302a expression in tumor tissues of patients with glioma (*N* = 40) and normal brain tissues (*N* = 10) determined by RT-qPCR. **C** Correlation between miR-302a expression and lncRNA HOXA-AS2 expression in glioma tissues (*N* = 40) analyzed by Pearson’s correlation coefficient. **D** miR-302a expression in 293 T cells transfected with miR-302a mimic determined by RT-qPCR. E, miR-302 binding to lncRNA HOXA-AS2 confirmed by dual-luciferase reporter gene assay in 293 T cells. **F** Binding sites of miR-302a on the 3′UTR of KDM2A mRNA predicted by the DIANA TOOLS, RAID v2.0 and TargetScan databases. **G** KDM2A expression in glioma tissues (*N* = 40) and normal brain tissues (*N* = 10) determined by RT-qPCR. **H** Correlation analysis between KDM2A expression and miR-302a expression in glioma tissues (*N* = 40) analyzed by Pearson’s correlation coefficient. **I** miR-302a binding to KDM2A validated by dual-luciferase reporter gene assay in 293 T cells. **J** Enrichment of KDM2A and lncRNA HOXA-AS2 analyzed by RIP in 293 T cells. **K** Expression of lncRNA HOXA-AS2, miR-302a and KDM2A in 293 T cells transfected with sh-lncRNA HOXA-AS2 or combined with miR-302a inhibitor determined by RT-qPCR. *p < 0.05. Data were shown as the mean ± standard deviation. Statistical comparisons were performed using unpaired *t*-test when only two groups were compared or by one-way ANOVA with Tukey’s post hoc test when more than two groups were compared. Cell experiments were repeated three times.

To dissect out the molecular mechanism by which lncRNA HOXA-AS2 affects glioma progression, we first predicted the direct downstream of lncRNA HOXA-AS2 using the online databases DIANA TOOLS, RAID v2.0, and TargetScan. The DIANA database predicted that miR-302a could bind to the 3′UTR of lncRNA HOXA-AS2 (Fig. 3A). RT-qPCR data presented lower miR-302a expression in glioma tissues than in normal brain tissues (Fig. 3B). Additionally, miR-302a expression showed a negative correlation with lncRNA HOXA-AS2 expression in glioma tissue samples (Fig. 3C). RT-qPCR results further revealed that miR-302a mimic remarkably augmented miR-302a expression in 293 T cells (Fig. 3D). Dual-luciferase reporter assay results suggested that the luciferase activity of lncRNA HOXA-AS2-WT was reduced in 293 T cells following transfection with miR-302a mimic, but that of lncRNA HOXA-AS2-MUT was not altered (Fig. 3E). Conclusively, miR-302a can bind to lncRNA HOXA-AS2.

Then, as predicted by the DIANA TOOLS, RAID v2.0 and TargetScan databases, miR-302a bound to the 3′UTR of KDM2A (Fig. 3F). Furthermore, RT-qPCR data depicted higher KDM2A expression in glioma tissues than in normal brain tissues (Fig. 3G). In addition, Pearson’s correlation coefficient analysis indicated the inverse correlation between KDM2A and miR-302a expression in the tumor samples (Fig. 3H). Meanwhile, miR-302a mimic inhibited the luciferase activity of KDM2A-WT but did not alter that of KDM2A-MUT in 293 T cells (Fig. 3I).

For further validation, RIP assay documented that the enrichment of lncRNA HOXA-AS2 was increased but that of KDM2A was reduced in the presence of lncRNA HOXA-AS2 overexpression (Fig. 3J), suggesting that lncRNA HOXA-AS2 and KDM2A could bind to miR-302a. Furthermore, lncRNA HOXA-AS2 silencing resulted in an elevation of miR-302a expression and a decline of lncRNA HOXA-AS2 and KDM2A expression. In contrast, simultaneous treatment with sh-lncRNA HOXA-AS2 and miR-302a inhibitor restrained miR-302a expression and enhanced KDM2A expression but did not affect lncRNA HOXA-AS2 expression relative to silencing of lncRNA HOXA-AS2 alone (Fig. 3K). In summary, lncRNA HOXA-AS2 could bind to miR-302a to upregulate KDM2A.

### LncRNA HOXA-AS2 induces glioma cell proliferation and immune tolerance to facilitate glioma progression by regulating the miR-302a/KDM2A axis

**Fig. 4:**
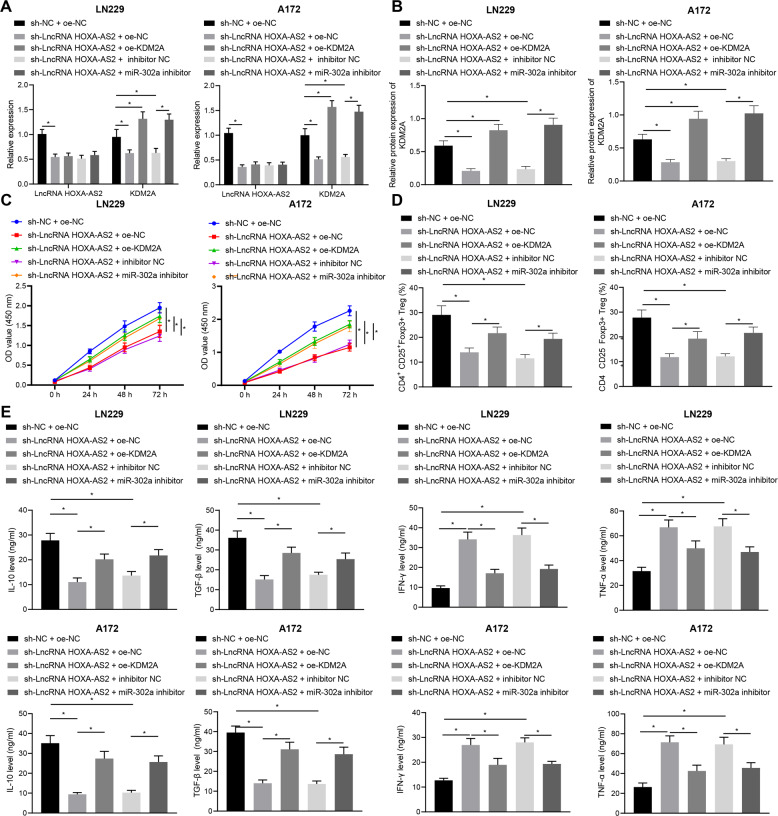
LncRNA HOXA-AS2 promotes T_reg_ cell proliferation and immune tolerance to facilitate glioma progression via the miR-302a/KDM2A axis. LN229 and A172 cells were treated with sh-NC + oe-NC, sh-lncRNA HOXA-AS2 + oe-NC, sh-lncRNA HOXA-AS2 + oe-KDM2A, and sh-lncRNA HOXA-AS2 + miR-302a inhibitor. **A** KDM2A expression in LN229 and A172 cells determined by RT-qPCR. **B** Western blot analysis of KDM2A protein in LN229 and A172 cells. **C** LN229 and A172 cell proliferation detected by CCK-8 assay. **D** Ratio of CD4^+^CD25^+^Foxp3^+^ cells in CD4^+^ cells co-cultured with LN229 and A172 cells analyzed by flow cytometry. **E** IL-10, TGF-β, IFN-γ, and TNF-α levels in the LN229 and A172 cell supernatant determined by ELISA. *p < 0.05. Data were shown as the mean ± standard deviation from. Statistical comparisons were performed using one-way ANOVA with Tukey’s post hoc test when more than two groups were compared. Variables were analyzed at different time points using two-way ANOVA with Bonferroni post hoc test. Cell experiments were repeated three times.

To further identify the function of lncRNA HOXA-AS2/miR-302a/KDM2A axis in glioma progression, we silenced lncRNA HOXA-AS2 using sh-lncRNA HOXA-AS2 and restored KDM2A expression using oe-KDM2A or repressed miR-302a using miR-302a inhibitor in LN229 and A172 cells. As reflected by RT-qPCR and Western blot analysis results, silencing of lncRNA HOXA-AS2 in LN229 and A172 cells triggered a reduction in lncRNA HOXA-AS2 and KDM2A expression, whilst further transfection with oe-KDM2A or miR-302a inhibitor elevated KDM2A expression without afflicting lncRNA HOXA-AS2 expression (Fig. 4A, B). CCK-8 assay indicated that LN229 and A172 cell viability was restricted upon lncRNA HOXA-AS2 silencing, which was nullified following further treatment with oe-KDM2A or miR-302a inhibitor (Fig. 4C). Furthermore, the LN229 and A172 cells treated as above were co-cultured with CD4^+^ cells. Here, oe-KDM2A or miR-302a inhibitor also counterweighed the decreased ratio of CD4^+^CD25^+^Foxp3^+^ cells caused by lncRNA HOXA-AS2 silencing (Fig. 4D). Consistent with this, our analysis of cytokine levels in supernatant from co-culture cells by ELISA showed that oe-KDM2A or miR-302a inhibitor annulled the reduction in IL-10 and TGF-β levels and the elevation in IFN-γ and TNF-α levels induced by sh-lncRNA HOXA-AS2 (Fig. 4E). Collectively, lncRNA HOXA-AS2 can promote glioma cell proliferation and immune tolerance to accelerate glioma progression by orchestrating the miR-302a/KDM2A axis.

### KDM2A facilitated JAG1 expression to promote T_reg_ cell proliferation and immune tolerance by increasing histone 3 lysine 4 (H3K4) tri-methylation (H3K4me3) modification

**Fig. 5:**
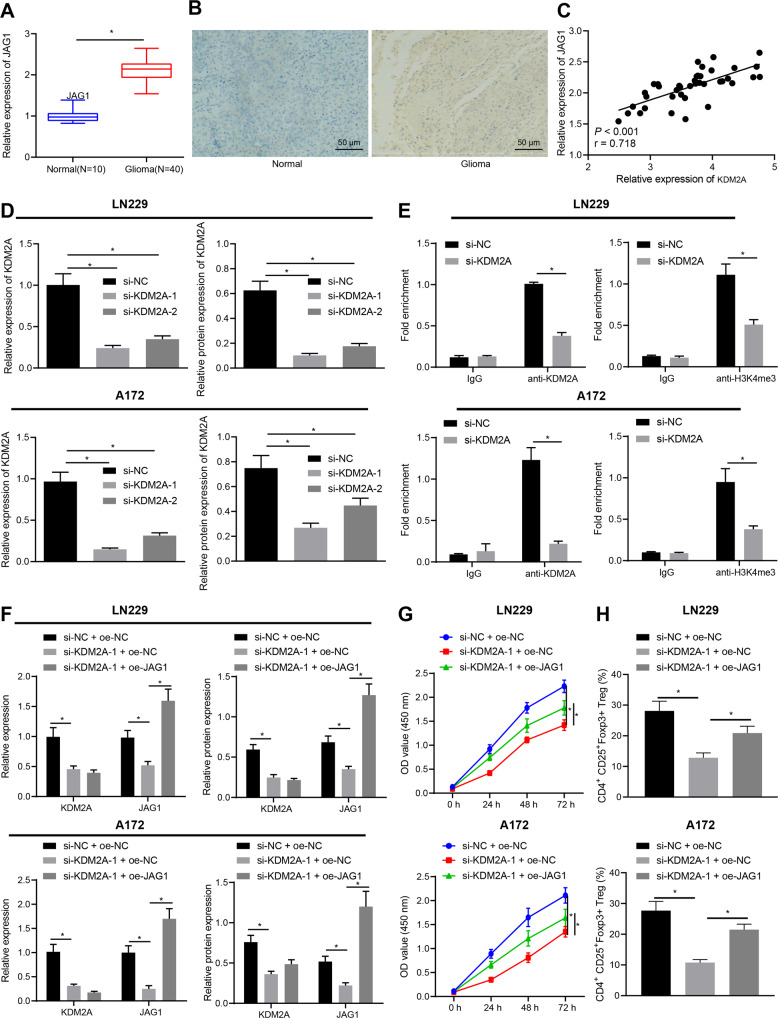
KDM2A promotes T_reg_ cell proliferation and glioma immune tolerance by upregulating JAG1 through affecting H3K4me3 in the JAG1 promoter. **A** JAG1 expression in glioma tissues (*N* = 40) and normal brain tissues (*N* = 10) determined by RT-qPCR. **B** JAG1 expression in glioma tissues (*N* = 40) and normal brain tissues (*N* = 10) detected by IHC. **C** Correlation analysis between KDM2A and JAG1 in glioma tissues (*N* = 40) using Pearson’s correlation coefficient. **D** KDM2A mRNA and protein expression in LN229 and A172 cells treated with si-KDM2A-1 and si-KDM2A-2 determined by RT-qPCR and Western blot analysis. **E** The enrichment of KDM2A and H3K4me3 on the JAG1 promoter determined by ChIP. LN229 and A172 cells were treated with si-NC + oe-NC, si-KDM2A-1 + oe-NC, or si-KDM2A-1 + oe-JAG1. **F** JAG1 and KDM2A mRNA and protein expression in LN229 and A172 cells determined by RT-qPCR and Western blot analysis. **G** LN229 and A172 cell proliferation detected by CCK-8 assay. **H** Ratio of CD4^+^CD25^+^Foxp3^+^ cells in CD4^+^ cells co-cultured with LN229 and A172 cells analyzed by flow cytometry. *p < 0.05. Data were shown as the mean ± standard deviation from. Statistical comparisons were performed using unpaired *t*-test when only two groups were compared or by one-way ANOVA with Tukey’s post hoc test when more than two groups were compared. Variables were analyzed at different time points using two-way ANOVA with Bonferroni post hoc test. Cell experiments were repeated three times.

Chen et al. reported that JAG1 was a direct downstream target of KDM2A in breast cancer [23]. RT-qPCR and IHC results presented that JAG1 mRNA and protein expression was high in glioma tissues (Fig. 5A, B) and shared a positive correlation with KDM2A expression in glioma tissues (Fig. 5C). RT-qPCR and Western blot analysis displayed that KDM2A expression was reduced in LN229 and A172 cells treated with si-KDM2A-1 and si-KDM2A-2, with si-KDM2A-1 presenting superior knockdown efficiency (Fig. 5D, Supplementary Fig. 1A) and was thus chosen for the following experimentation.

Next, ChIP assay manifested that both KDM2A and H3K4me3 could bind to the promoter region of JAG1, whereas the enrichment of KDM2A and H3K4me3 at the promoter region of JAG1 was considerably after KDM2A silencing (Fig. 5E). As displayed in Fig. 5F and Supplementary Fig. 1B, RT-qPCR and Western blot analysis illustrated reduced JAG1 expression in LN229 and A172 cells silencing KDM2A while further overexpression of JAG1 elevated JAG1 expression without altering that of KDM2A. Taken together, KDM2A could upregulate JAG1 by promoting the methylation modification of the JAG1 promoter region.

Furthermore, CCK-8 assay results illustrated that overexpressing JAG1 rescued LN229 and A172 cell viability suppressed by KDM2A silencing (Fig. 5G). The LN229 and A172 cells treated as above were co-cultured with CD4^+^ cells, which depicted that JAG1 overexpression also perfectly counteracted the reduced ratio of CD4^+^CD25^+^Foxp3^+^ cells caused by KDM2A silencing (Fig. 5H). Collectively, KDM2A elevated JAG1 expression by promoting the methylation modification of the JAG1 promoter region, thus inducing T_reg_ cell proliferation and immune tolerance.

### LncRNA HOXA-AS2 promoted glioma progression by manipulating the miR-302a-KDM2A-JAG1 axis in vivo

**Fig. 6:**
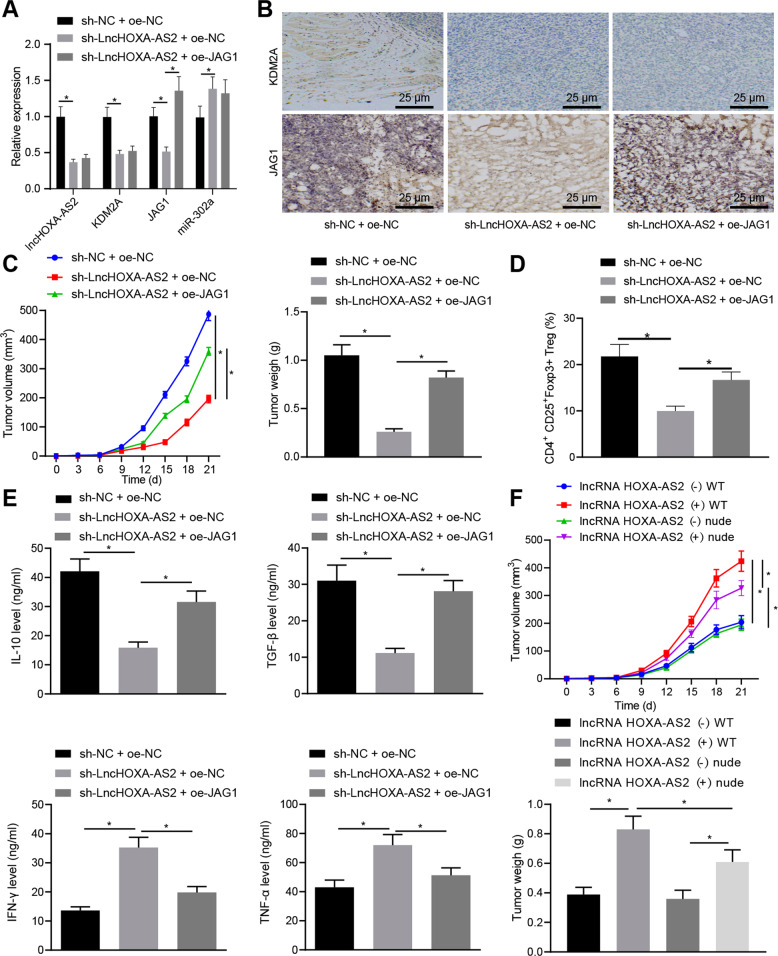
LncRNA HOXA-AS2 accelerates glioma progression in vivo via the miR-302a/KDM2A/JAG1 axis. BALB/c mice were injected with LN229 cell suspension stably infected with lentivirus harboring sh-NC + oe-NC, sh-lncRNA HOXA-AS2 + oe-NC or sh-lncRNA HOXA-AS2 + oe-JAG1. **A** LncRNA HOXA-AS2, KDM2A, JAG1, and miR-302a expression in tumor tissues of BALB/c mice determined by RT-qPCR. **B** KDM2A and JAG1 expression in tumor tissues of BALB/c mice determined by IHC. **C** Measurement of tumor volume and weight of BALB/c mice. **D** Ratio of CD4^+^CD25^+^Foxp3^+^ cells in tumor tissues of BALB/c mice analyzed by flow cytometry. **E** IL-10, TGF-β, IFN-γ, and TNF-α levels in the BALB/c mouse spleen tissues determined by ELISA. **F** The tumor volume and weight of BALB/c mice and nude mice. WT indicates normal BALB/c mice and nude indicates immunocompromised nude mice. *p < 0.05. Data were shown as the mean ± standard deviation. Statistical comparisons were performed using one-way ANOVA with Tukey’s post hoc test when more than two groups were compared. Variables were analyzed at different time points using Bonferroni-corrected repeated measures ANOVA. *n* = 8.

To investigate the role of the lncRNA HOXA-AS2/miR-302a/KDM2A/JAG1 axis in glioma progression, we established a xenograft mouse model, in conjunction with lncRNA HOXA-AS2 silencing and JAG1 overexpression. RT-qPCR results documented that lncRNA HOXA-AS2, KDM2A, and JAG1 expression was diminished, while that of miR-302a was augmented in tumor tissues of BALB/c mice silencing lncRNA HOXA-AS2. However, upregulated JAG1 was observed in tumor tissues of BALB/c mice treated with oe-JAG1 along with no significant alterations in the lncRNA HOXA-AS2, KDM2A and miR-302a expression in the presence of sh-lncRNA HOXA-AS2 (Fig. 6A). IHC exhibited that lncRNA HOXA-AS2 silencing potently reduced KDM2A and JAG1 expression in tumor tissues of BALB/c mice, whereas JAG1 overexpression triggered the elevation in JAG1 expression in tumor tissues of BALB/c mice without impacting did KDM2A expression in the presence of lncRNA HOXA-AS2 silencing (Fig. 6B).

Furthermore, tumor volume and weight of BALB/c mice were lowered in response to lncRNA HOXA-AS2 silencing, while further JAG1 overexpression abrogated these trends (Fig. 6C). Consistent with this result, JAG1 upregulation also negated the decreased ratio of CD4^+^CD25^+^Foxp3^+^ cells in tumor tissues caused by lncRNA HOXA-AS2 silencing (Fig. 6D). Meanwhile, upregulation of JAG1 counterweighed the downregulated IL-10 and TGF-β and the upregulated IFN-γ and TNF-α triggered by lncRNA HOXA-AS2 silencing (Fig. 6E).

Furthermore, the tumor volume and weight of lncRNA HOXA-AS2 (+) WT mice were greater than that of lncRNA HOXA-AS2 (−) WT mice. In addition, the tumor volume and weight of lncRNA HOXA-AS2 (+) nude mice were reduced relative to lncRNA HOXA-AS2 (+) WT mice. Compared to the lncRNA HOXA-AS2 (+) nude mice, the lncRNA HOXA-AS2 (−) nude mice showed diminished tumor volume and weight (Fig. 6F). These data demonstrated that lncRNA HOXA-AS2 might accelerate the proliferation and immune tolerance of glioma cells, thereby promoting the growth of glioma. Taken together, our study demonstrated that lncRNA HOXA-AS2 promoted glioma progression in vivo by mediating the miR-302a/KDM2A/JAG1 axis.

## Discussion

T_reg_ cells include two main types, namely the natural T regulatory (nT_reg_) cells originating in the thymus, and the inducible T regulatory (iT_reg_) cells that are typically induced upon exposure to antigens in a tolerogenic environment; both of these T_reg_ cell types can promote glioma-mediated immune suppression [24]. Accumulated evidence reveals that T_reg_ cells inhibit immune response by secreting several cytokines, including TGF-β and IL-10 [25]. Recently, lncRNAs have emerged as important regulators for tumor immune response [11]. In this study, our investigations revealed that lncRNA HOXA-AS2 was significantly upregulated both in glioma biopsy specimens and in glioma cell lines. Furthermore, in vivo data demonstrated that lncRNA HOXA-AS2 silencing suppressed T_reg_ cell proliferation, which resulted in reduced secretion of TGF-β and IL-10 in spleen and finally reduced immune tolerance in glioma.

Our findings suggested that lncRNA HOXA-AS2 was upregulated in glioma, whereas its silencing could suppress the tumor growth. Consistent with this result, a previous study has revealed that lncRNA HOXA-AS2 is upregulated in glioma [26]. In addition, lncRNA HOXA-AS2 knockdown in osteosarcoma has been demonstrated to attenuate xenograft tumor growth [27]. Also, a recent study has revealed the oncogenic role of lncRNA HOXA-AS2 in papillary thyroid cancer [28]. In addition to its oncogenic role, our data provided more mechanistic evidence for the concept that HOXA-AS2 silencing could repress T_reg_ cell proliferation and immune tolerance. CD4^+^ cells are T lymphocytes expressing CD4 antigen, the main function of which is to assist CD8^+^ T lymphocytes and participate in the body’s cellular immune response [29]. The transcription factor Foxp3 is specifically expressed in T_reg_ cells and is closely related to the growth, development, and function maintenance of T_reg_ cells [30]. In addition, the ratio of CD4^+^CD25^+^Foxp3^+^ cells may be correlated with immune tolerance and tumor cell survival [31]. Intriguingly, our data illustrated that lncRNA HOXA-AS2 silencing diminished the ratio of CD4^+^CD25^+^Foxp3^+^ cells.

Previous studies have demonstrated that lncRNAs exert their effects in regulating gene expression by acting as scaffolds for epigenetic factors or by competitively binding to target miRNAs [14, 19]. A recent study has identified that lncRNA HOXA-AS2 competitively binds to miR-520a-3p and hence facilitates NSCLC progression [32]. By performing dual-luciferase reporter and RIP assays, we found that lncRNA HOXA-AS2 could competitively bind to miR-520a-3p. Various miRNAs have already been reported to play a role in tumor-mediated immune suppression [33]. For instance, miR-124 can promote T cell-mediated immune clearance of glioma by suppressing signal transducer and activator of transcription 3 (STAT3) signaling [34]. miRNAs are also reported to regulate cervical cancer progression by inducing Th17/T_reg_ imbalance [35]. Accumulated evidence has showed that miR-302a act as a tumor suppressor in diverse kinds of cancers, including colorectal cancer and hepatocellular carcinoma [36–38]. Consistent with previous data, we found that miR-302a expression was significantly downregulated in our glioma patient samples and glioma cell lines. Notably, miR-302a could reduce glioma-induced immune tolerance mediated by lncRNA HOXA-AS2.

Another finding of this study was that KDM2A was a target gene of miR-302a and that lncRNA HOXA-AS2 upregulated the KDM2A expression by attenuating the binding of miR-302a to KDM2A. KDM2A is a histone demethylase, which has been reported to demethylate specifically histone H3 at lysine 36 [39]. Also, H3K4me3 has been verified to be a new direct substrate of KDM2A [40]. Abundant data have revealed that KDM2A acts as an oncogene to promote the progression of various cancers, including glioma [41–43]. KDM2A silencing has been shown to inhibit the proliferation, migration and invasion of glioma cells, suggesting the oncogenic role of KDM2A in the progression of glioma [41]. In our study, we found that KDM2A expression was elevated in glioma biopsy specimens and glioma cell lines and that lncRNA HOXA-AS2 positively regulated the expression of KDM2A to promote glioma cell proliferation and immune tolerance.

JAG1 is a known direct downstream target of KDM2A, which was reported to be upregulated by KDM2A through removal of the tri-methylation of histone H3 lysine 36 in its promoter [23]. As one of the most studied factors in the Notch signaling pathway, JAG1 is known to function as tumor promoter in malignancies by inducing angiogenesis or immune cell infiltration [44, 45]. Accumulating evidence has highlighted the involvement of JAG1 in the regulation of immune suppression *via* several different pathways, including by controlling T_reg_ cells [46, 47]. Those findings have suggested that the formation of JAG-1-conjugated islets leads to a significant increase of T_reg_ cell proliferation in the tumor. In our study, we substantiated that KDM2A could specifically promote the JAG1 expression by recruiting H3K4me3, thereby promoting immune tolerance.

**Fig. 7:**
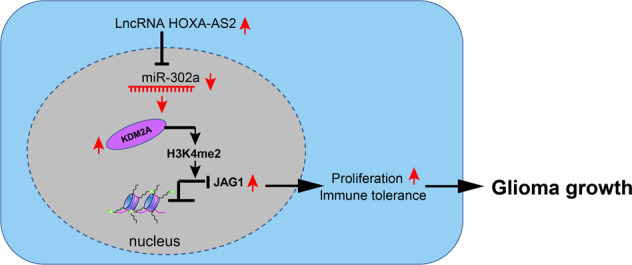
Schematic diagram of the mechanism by which lncRNA HOXA-AS2 affects glioma progression. LncRNA HOXA-AS2 is abundantly expressed in glioma. It competitively binds to miR-302a and causes its downregulation, thus weakening its binding to KDM2A, the target gene of miR-302a. By this mechanism, the expression of KDM2A is enhanced, which promotes JAG1 promoter methylation, thus accelerating glioma cell and T_reg_ cell proliferation and glioma cell immune tolerance, ultimately promoting the progression of glioma.

In summary, our investigation revealed that lncRNA HOXA-AS2 upregulated KDM2A expression and promoted JAG1 expression in glioma cells by competitively binding to miR-302a (Fig. 7). Results of function analyses in vivo and in vitro demonstrated that lncRNA HOXA-AS2-miR-302a-KDM2A-JAG1 axis was essential for immune tolerance in glioma by regulating T_reg_ cell proliferation. Our study not only uncovered a novel molecular mechanism for glioma progression, but also provided a therapy target for glioma. However, it remains to be established how the lncRNA HOXA-AS2-miR-302a-KDM2A-JAG1 axis regulates T_reg_ cell proliferation, considering that subcutaneous injection of the tumor cells does not properly model glioma with respect to anatomic location. Obtaining better knowledge of mechanisms underlying glioma tolerance is essential precondition for targeted glioma therapy. Also, more studies are necessary to investigate the effect of lncRNA HOXA-AS2 silencing on other aspects of T_reg_ cell biology.

## Materials and methods

### Ethics statement

The current study was performed with the approval of the Ethics Committee of the Affiliated Hospital of Southwest Medical University (Luzhou, Sichuan, China) and performed in strict accordance with the *Declaration of Helsinki*. All participants signed informed consent documentation. Animal experiments were approved by the Animal Care and Use Committee of the Affiliated Hospital of Southwest Medical University and performed in strict accordance to the Guide for the Care and Use of Laboratory Animals published by the US National Institutes of Health. Extensive efforts were made to ensure minimal suffering of the included animals.

### Microarray-based gene expression profiling

Glioma-related mRNA expression dataset GSE15824 was retrieved from the Gene Expression Omnibus database. GSE15824 dataset contained two normal samples and twelve tumor samples. Differential analysis was conducted with normal samples as a control. Differentially expressed genes (DEGs) were identified using R language “limma” package (version 3.4.1) [48]. After several tests by Benjamini and Hochberg, |log2FC | > 0.5 and adjusted *p* < 0.05 (positive false discovery rate < 0.05) were utilized as the criteria for screening DEGs. The expression of DEGs was analyzed in glioma using the UALCAN website [49].

### Study subjects

Cancer tissues were attained from 40 patients with glioma who underwent surgical treatment at the Department of Neurosurgery of the Affiliated Hospital of Southwest Medical University (Luzhou, Sichuan, China) and normal brain tissues of ten patients undergoing craniocerebral trauma surgery at the Affiliated Hospital of Southwest Medical University (Luzhou, Sichuan, China) were selected as controls. The harvested tissue samples are stored in liquid nitrogen.

### Cell culture

Human glioma cell lines LN229, A172, and T98G, normal human glial cell line Heb and normal human renal epithelial cells (293T) were acquired from American Type Culture Collection (Manassas, VA, USA). Human glioma cell line U373-MAGI was attained from Wuhan Sunma Biotechnology Corp. (Wuhan, Hubei, China). All cells were within passage 30. The LN229 and A172 cell lines are astrocytoma cells. All cells were cultured in Dulbecco’s modified Eagle’s medium (Sigma-Aldrich; Merck KGaA, Darmstadt, Germany) encompassing 10% fetal bovine serum (FBS; Gibco; Thermo Fisher Scientific Inc., Waltham, MA, USA), 100 U/mL penicillin and 100 µg/mL streptomycin (Sigma-Aldrich; Merck KGaA) and maintained at 37 °C in a wet environment encompassing 5% CO_2_.

### Cell treatment

Cells were subjected to no treatment (control) or were transfected with vectors for short hairpin RNA-negative control (sh-NC), sh-HOXA-AS2-1, sh-HOXA-AS2-2, overexpression (oe)-NC, oe-HOXA-AS2, mimic-NC, miR-302a mimic, sh-NC + inhibitor NC, sh-HOXA-AS2 + miR-302a inhibitor, sh-NC + oe-NC, sh-HOXA-AS2 + oe-NC, sh-HOXA-AS2 + oe-lysine demethylase 2 A (KDM2A), small interfering RNA (si)-NC, si-KDMA2A-1, si-KDMA2A-2, si-NC + oe-NC, or si-KDMA2A-1 + oe-JAG1.

The vector used for shRNAs was pLVshRNA-EGFP(2A)puro, and the overexpression vector was pLVX-puro (RiboBio, Guangdong, China). sh-HOXA-AS2-1, sh-HOXA-AS2-2, oe-HOXA-AS2, miR-302a mimic, miR-302a inhibitor, oe-KDM2A, si-KDMA2A, si-KDMA2A-2, and oe-JAG1 were all purchased from RiboBio. The specific cell transfection procedures were as follows: the cells were initially seeded into a 50 mL culture flask with complete medium and cultured until they reached 50–60% confluence. Next, Lipofectamin 2000 (Gibco) and RNA or DNA to be transfected were prepared in a sterile Eppendorf (EP) tube. Specifically, 5 μL Lipofectamin 2000 was mixed with 100 μL serum-free medium and allowed to stand at room temperature for 5 min. The RNA (50 nmol) or DNA (2 μg) to be transferred were mixed with 100 μL serum-free medium and left to stand at room temperature for 20 min to allow formation of the complex of RNA or DNA with liposomes. The cells in the culture flask were then washed with serum-free medium, and serum-free culture medium (without antibiotics) was added to the complex and gently mixed. The mixture was transferred into a 10 mL culture bottle for transfection, which was placed in a 5% CO_2_ incubator at 37 °C, followed by complete medium renewal after 6–8 h. The sequences used are depicted in Supplementary Table 1.

### CCK-8 assay

A CCK-8 kit (Dojindo Laboratories, Kumamoto, Japan) was employed. In brief, glioma cells were subjected to detachment and seeding in 96-well plates at 2 × 10^3^ cells/well. Subsequent to 48 h culture, 10 µL CCK-8 solution was supplemented into each well for 2 h incubation with the cells at 37 °C, whereupon the absorbance was measured at 450 nm. Cell viability rate was calculated as the cell viability of the experimental group divided by cell viability of the control group × 100%.

### Reverse transcription quantitative polymerase chain reaction (RT-qPCR)

Following 24 h transduction, total RNA was extracted using the Roche RNA isolation kit (Indianapolis, IN, USA). Complementary DNA (cDNA) from mRNA was generated following the manuals of a commercially available kit (RR047A, Takara, Japan). cDNA for miRNA detection was synthetized using a commercial miRcute miRNA first-strain synthesis kit (Tiangen Biotech, Beijing, China). Real-time PCR was implemented using the GoTaq^®^ two-Step RT-qPCR System (Promega, Madison, WI, USA) in an ABI Prism 7500 instrument (Applied Biosystems). miR-302a expression was normalized to U6 and that of the remaining mRNAs was normalized to glyceraldehyde-3-phosphate dehydrogenase (GAPDH). The fold changes were calculated using the 2^−△△CT^ method. Indicated primers are listed in Supplementary Table 2.

### Immunoblotting

Cells were collected by detachment with trypsin and then lysed in radio immunoprecipitation assay lysis buffer (Boster, Hubei, China), followed by estimation of protein concentration using a bicinchoninic acid quantification kit (Boster). Subsequent to separation using freshly-prepared 10% sodium dodecyl sulfate (SDS)-polyacrylamide gel electrophoresis, the protein was electro-transferred onto polyvinylidene fluoride membranes. The membrane underwent 5% bovine serum albumin (BSA) blocking and probing with primary antibodies (Abcam, Cambridge, UK) to KDM2A (ab191387, 1:1000), JAG1 (ab7771, 1:500) and GAPDH (ab181602, 1:500). Following incubation with secondary antibody goat anti-rabbit immunoglobulin G (IgG) (ab205719, 1:500, Abcam), immunoblots were visualized with enhanced chemiluminescence detection reagents (Millipore Corp., Bedford, MA, USA) and then captured under the Bio-Rad image system (Bio-Rad, Hercules, CA, USA). Gray values of target protein bands were quantified using Image J software, with GAPDH as a normalizer.

### Dual-luciferase reporter gene assay

The binding sites between KDM2A and miR-302a, as well as between HOXA-AS2 and miR-302a were predicted. Wild type (WT) or mutant type (MUT) KDM2A 3′untranslated region (3′UTR) and lncRNA HOXA-AS2 sequences were cloned into pmirGLO plasmid (Inovogen Tech. Co., Ltd., Chongqing, China). The indicated pmirGLO constructs were co-transfected into 293 T cells with miR-302a or mimic NC for 24 h with Lipofectamine^™^ 2000 reagent, and luciferase activity was measured using the Dual-Luciferase Reporter Gene Assay System (Promega) in the light of the manufacturer’s instructions.

### RNA binding protein immunoprecipitation (RIP) assay

Lysates were attained after cells transfected with si-NC or si-lncRNA HOXA-AS2 and incubated with Argonaute2 (Ago2) antibody (Millipore). RIP was implemented in the light of protocols of a Magna RIP TM RNA binding protein immunoprecipitation kit (Millipore). Separated RNA from immunoprecipitation was quantified by nano-spectrophotometry (Implen, Munich, Germany). LncRNA HOXA-AS2 and KDM2A expression was determined by RT-qPCR.

### Chromatin immunoprecipitation (ChIP)

Subsequent to 10 min 1% formaldehyde fixing, cells underwent two washes with phosphate buffer saline (PBS) encompassing protease inhibitors (1 mM benzenesulfonylfluoride, 1 μg/mL aprotinin and 1 μg/mL pepsin A), 10 min culture in lysis buffer (1% SDS, 10 mM ethylenediaminetetraacetic acid [EDTA], 50 mM Tris-HCl, pH = 8.1), and sonication. Following 10 min lysate centrifugation at 13,000 *r/min*, the supernatant was diluted by dilution buffer (0.01% SDS, 1% Triton X-100, 2 mM EDTA, 16.7 mM Tris-HCl pH = 8.1, 167 mM NaCl and protease inhibitors) and incubated with antibody to IgG, KDM2A (1:80, ab191387, Abcam), or H3K4 (1:100, ab8580, Abcam) at 4 °C. DNA fragments were collected and amplified by PCR with specific primers.

### Enzyme-linked immunosorbent assay (ELISA)

Interleukin-10 (IL-10) (M1000B), tumor necrosis factor-α (TNF-α) (DTA00D), interferon-gamma (INF-λ) (DIF50C), and transforming growth factor-β (TGF-β) (DG100B) ELISA kits were acquired from R&D Systems Inc. (Minneapolis, Minn., USA). All reagents, standards and experimental samples (experimental samples were spleen grinding fluid) were prepared in the light of the manufacturer’s protocols. Then, 100 µL gradient diluted samples were supplemented to the plate for 2 h incubation at ambient temperature. After incubation, samples were incubated with 200 µL related antibody at room temperature for 2 h. The 200 µL portions of substrates were added for 30 min incubation in a dark room. Finally, 50 µL stop buffer was supplemented to arrest the reaction, and the absorbance was measured at 450 nm.

### Tumor xenograft experiment

Seventy-two male BALB/c mice (4 weeks old, weighing 18–25 g) from Hunan SJA Laboratory Animal Co., Ltd. (Human, China) were housed in a specific pathogen-free environment. The mice were randomly arranged into nine groups (eight mice per group) for subsequent experiments. Next, the mice were subcutaneously inoculated with LN229 and A172 cells stably transfected with sh-NC or sh-lncRNA HOXA-AS2, or LN229 cells with lncRNA HOXA-AS2 (−) WT, lncRNA HOXA-AS2 (+) WT, sh-NC + oe-NC, sh-lncRNA HOXA-AS2 + oe-NC and sh-lncRNA HOXA-AS2 + oe-JAG at a density of 1 × 10^7^ cells/mouse. Upon reaching 80% confluence, cells were counted and resuspended in PBS. The cell suspension (5 × 10^6^ cells) was subcutaneously injected into each mouse. Bidimensional tumor measurement (the maximum diameter and minimum diameter for each tumor) was recorded using Vernier calipers every 3–4 days. Tumor volume of the nude mice was calculated using the formula: tumor volume = 0.5 × ab^2^, where a is the maximum diameter and b is the minimum diameter. The maximum diameter of tumors was 0.79 ± 0.06 cm in the sh-NC + oe-NC group. The health condition and behaviors of the nude mice were monitored on a daily basis. At 21 days after tumor cell inoculation, the nude mice were euthanized by cervical dislocation and tumor tissues were excised, followed by tumor weight measurement. The largest tumors weighed 1.05 ± 0.11 g with a volume <1000 mm^3^. The tumor tissues were cut into slices for subsequent immunohistochemistry (IHC) processing.

Sixteen nude mice (4 weeks old, weighing 18–25 g) from Hunan SJA Laboratory Animal Co., Ltd. were housed in a specific pathogen-free environment. The mice were randomly assigned into two groups (eight mice per group): lncRNA HOXA-AS2 (−) [nude mice were inoculated with LN229 cells with lncRNA HOXA-AS2 (−)] and lncRNA HOXA-AS2 (+) [nude mice were inoculated with LN229 cells with lncRNA HOXA-AS2 (+)]. Cell inoculation for the nude mice was carried out according to the procedures described above.

### IHC

Subsequent to formalin fixing and paraffin embedding, 5 μm tissue slices were deparaffinized in xylene and rehydrated through a gradient series of alcohols. Following antigen retrieval, all slices were blocked at ambient temperature in avidin/biotin blocking buffer (C-0005, HaoRan Biotech Co., Ltd., Shanghai, China) and then with 3% BSA for 30 min. Following that, the slices were immunostained with primary rabbit antibodies (Abcam) to KDM2A (ab191387, 1:1000), JAG1 (ab7771, 1:500), FAPα (#ab53066, 1:100), CD4 (ab183685, 1:1000), T-bet (ab150440, 1:1000), Foxp3 (#ab20034, 1:500) and CD25 (ab231441, 1:100) at 4 °C overnight. The slices underwent 20 min incubation with secondary goat anti-rabbit IgG (ab6785, 1:1000, Abcam) at 37 °C. Next, the slices were added with streptomycin ovalbumin working solution tagged with horseradish peroxidase (0343-10000U, Yimo Biotechnology Co., Ltd., Beijing, China) and positioned at 37 °C for 20 min, followed by developing with a diaminobenzidine substrate kit (ST033; Guangzhou Weijia Technology Co., Ltd., Guangdong, China). The slices received 1 min counterstaining with hematoxylin (PT001, Bogoo, Shanghai, China). Afterwards, the slices were dehydrated by an ethanol concentration gradient, cleared, and sealed by neutral resin. IHC images were obtained using an upright microscope. Five high power fields of view containing at least 100 cells were chosen from each section. The excised tumors were identified as astrocytoma by experienced pathologists blinded to the experimental groups.

### Flow cytometry

Cells were lysed and centrifuged. With the removal of the supernatant, the pellets were rinsed thrice by 0.5% BSA. Afterwards, the cells were incubated with antibodies (Abcam) to CD4 (PE) (ab18282), and Foxp3 (Fluor488) (ab187598, 1:100) for 30 min in the dark. The supernatant was removed following 5 min centrifugation at 350 × *g*, and the pellets were rinsed three times with 0.5% BSA. With the removal of the supernatant, the pellets were resuspended in 200 μL 0.5% BSA for further analysis. To prepare CD4^+^CD25^+^ T_reg_ cells, the isolated CD4^+^ T cells were stained with anti-nude mouse allophycocyanin (APC)-conjugated CD25 antibody (ab25485, Abcam), followed by positive selection through autoMACS using anti-APC microbeads. The BD LSRII cell sorter (Sony, Tokyo, Japan) was employed to sort target cells.

### Tumor immune cell isolation

The tumor was removed, and cut into pieces. The tissue pieces were detached by addition of 1 mg/mL collagenase D (Roche Life Science, Basel, Switzerland) and 100 mg/mL DNase I (Roche) for 1 h at ambient temperature. After detachment, the samples were passed through a 70 µm nylon mesh filter to remove debris and epithelial cells. T_reg_ cells were counted using flow cytometry.

### CD4^+^ T cell isolation from nude mice and T_reg_ cell detection

On the 18th day after implantation of tumor cells, nude mice were euthanized and tissues (tumor) were excised and placed in a 60 cm Petri dish encompassing 3 mL Dulbecco’s PBS. A 40 µm sterile nylon filter was placed on the tissues using sterile tweezers. The tissues were gently ground and dispersed into a suspension. A new square nylon filter was placed on the opening of a 15 mL cone-shaped tube, and the suspension was filtered to remove the remaining tissue fragments. The mouse tumor and cell suspension was prepared using a CD4^+^ T cell isolation kit and autoMACS Pro (Miltenyi Biotec, Bergisch Gladbach, Germany). The CD4^+^ T cells were isolated using antibodies against CD4, CD25 and homoantibodies, centrifuged, and then fixed with paraformaldehyde to reselect the cells.

Flow cytometry detection was subsequently conducted. The ratio of Foxp3+ cells in CD4^+^ cells was analyzed by setting CD4^+^ cells as the gate, whereas the lymphocyte gate was R1. Analysis of the cells in the R1 gate showed that the CD4^+^ cells were R2. Next, the results of T_reg_ were obtained in their respective R2, and the proportion of T_reg_ cells was expressed as the percentage of CD4^+^CD25^+^Foxp3^+^ in CD4^+^ T cells.

### Statistical analysis

All data were processed using SPSS 21.0 statistical software (IBM Corp., Armonk, NY, USA). Data were shown as the mean ± standard deviation. Data obeying normal distribution and homogeneity of variance between two groups were compared using unpaired *t*-test. Data among multiple groups were assessed by one-way analysis of variance (ANOVA), followed by Tukey’s post hoc tests with corrections for multiple comparisons. Data of tumor volume at different time points were compared by repeated measures ANOVA with Bonferroni post hoc tests, and those of OD values at different time points were compared by two-way ANOVA. Correlation analysis was performed using Pearson’s correlation coefficient. Survival rate was calculated by the Kaplan–Meier method. *p* < 0.05 was considered as a level of statistical significance.

## Supplementary information

Supplementary Materials

aj-checklist

## Data Availability

Data required to support the findings of this study are present in the main text or supplementary materials. All other data supporting the findings of this study are available from the corresponding authors upon request.
