# Supplementary material for: HOXA-AS2 contributes to regulatory T cell proliferation and immune tolerance in glioma through the miR-302a/KDM2A/JAG1 axis
Source: Cell Death Dis. 2022 Feb 18;13(2):160. doi: 10.1038/s41419-021-04471-4 (PMC8857186; doi:10.1038/s41419-021-04471-4)
Supplement: Supplementary file 1 — Supplementary Materials [file 41419_2021_4471_MOESM1_ESM.docx]

**Supplementary Fig. 1. Representative Western blots of panels 5D (A), and 5F (B).**


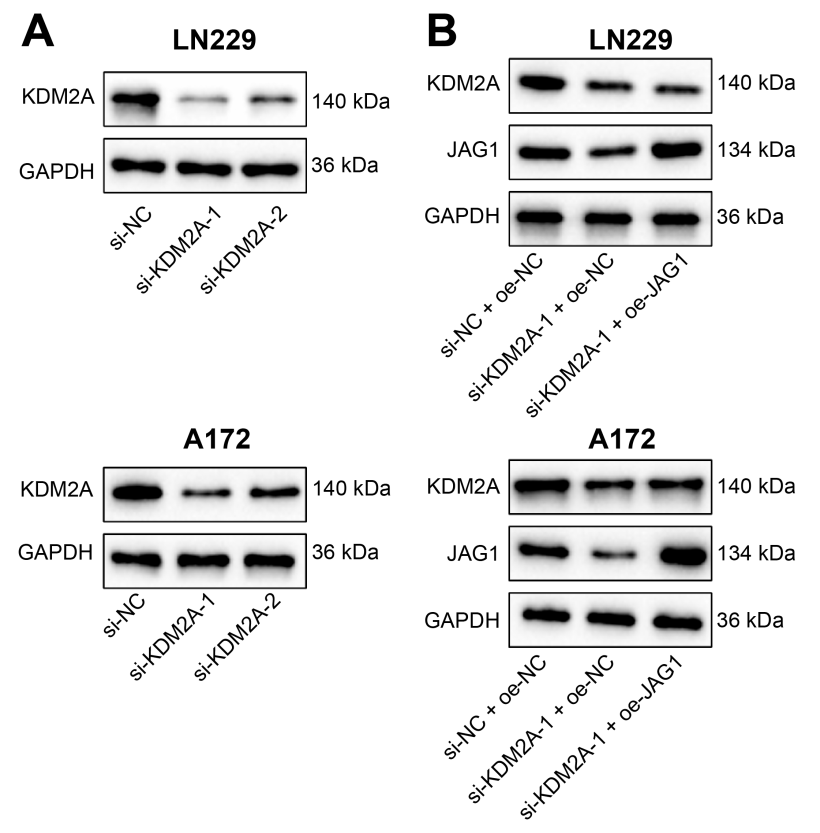


**Supplementary Table 1.** Oligonucleotide sequences for transfection

| Vector | Sequence |
| --- | --- |
| sh-HOXA-AS2-1 | 5'-AAACCTTGTAGATAGCTTGAGCTGG-3' |
| sh- HOXA-AS2-2 | 5'-GAGTTCAGCTCAAGTTGAACATACA-3' |
| si-KDM2A-1 | 5'-TTCTGGAATCTCCCAGAGCAC-3' |
| si-KDM2A-2 | 5'-TATTCTGGAATCTCCCAGAGC-3' |
| miR-302a mimic | 5'-CAAAACGUGAGGCGCUGCUAU-3' |
| miR-302a inhibitor | 5'-GUUUUGCACUCCGCGACGAUA-3' |

**Supplementary Table 2.** Primer sequences used for RT-qPCR

| Gene | Sequence |
| --- | --- |
| HOXA-AS2 | F: 5'-TGAACCAGGAATTGTCTCCA-3'  R: 5'-CATCTCCCACTCCCAGAAAG-3' |
| GAPDH | F: 5'-GTGGACCTGACCTGCCGTCT-3'  R: 5'-GGAGGAGTGGGTGTCGCTGT-3' |
| miR-302-3p | F: 5'-TAAGTGCTTCCATGTTTTGGTGA-3'  R: 5'-GAACATGTCTGCGTATCTCAGACTTC-3' |
| JAG1 | F: 5'-GACTCATCAGCCGTGTCTCA-3'  R: 5'-TGGGGAACACTCACACTCAA-3' |
| U6 | F: 5'-ATTGGAACGATACAGAGAAGATT-3'  R: 5'-GGAACGCTTCACGAATTTG-3' |
| KDM2A | F: 5'-GTGACGCAGCAGCATTGTTCATTGTTC-3'  R: 5'-GCAGAGACTGCAGACCAGGAGCA-3' |
| hsa-miR-302a | F: 5'-UAAGUGCUUCCAUGUUUUGGUGA-3'  R: Universal primer |

Notes: RT-qPCR, reverse transcription-quantitative polymerase chain reaction; F, forward; R, reverse; HOXA-AS2, homeobox A cluster antisense RNA 2; GAPDH, glyceraldehyde-3-phosphate dehydrogenase; miR-302a-3p, microRNA-302a-3p; JAG1, Jagged-1; KDM2A, lysine demethylase 2A
